# Investing in the health workforce: fiscal space analysis of 20 countries in East and Southern Africa, 2021–2026

**DOI:** 10.1136/bmjgh-2021-008416

**Published:** 2022-06-30

**Authors:** James Avoka Asamani, Jesse Kigozi, Brivine Sikapande, Christmal Dela Christmals, Sunny C Okoroafor, Hamza Ismaila, Adam Ahmat, Jennifer Nyoni, Juliet Nabyonga-Orem, Kasonde Mwinga

**Affiliations:** 1 Health Workforce Unit, Universal Health Coverage - Life Course Cluster, World Health Organization Regional Office for Africa, Brazzaville, Congo; 2 Faculty of Health Sciences, Centre for Health Professions Education, North-West University - Potchefstroom Campus, Potchefstroom, South Africa; 3 Health Economics Unit, University of Birmingham, Birmingham, UK; 4 Monitoring and Evaluation Unit, Zambia Ministry of Health, Lusaka, Zambia; 5 Faculty of Health Sciences, Centre for Health Professions Education, North-West University, Potchefstroom, South Africa; 6 Office of the Director-General, Headquarters, Ghana Health Service, Accra, Ghana; 7 Health Financing and Investment Unit, Universal Health Coverage - Life Course Cluster, World Health Organization Regional Office for Africa, Brazzaville, Congo; 8 Faculty of Health Sciences, Center for Health Professions Education, North-West University, Potchefstroom, South Africa; 9 Universal Health Coverage - Life Course Cluster, World Health Organization Regional Office for Africa, Brazzaville, Congo

**Keywords:** mathematical modelling, health economics, health policy, health systems, health services research

## Abstract

**Background and objectives:**

The health workforce (HWF) is at the core of ensuring an efficient, effective and functional health system, but it faces chronic underinvestment. This paper presents a fiscal space analysis of 20 countries in East and Southern Africa to generate sustained evidence-based advocacy for significant and smarter investment in the HWF.

**Methods:**

We adapted an established empirical framework for fiscal space analysis and applied it to the HWF. Country-specific data were curated and triangulated from publicly available datasets and government reports to model the fiscal space for the HWF for each country. Based on the current knowledge, three scenarios (business as-usual, optimistic and very optimistic) were modelled and compared.

**Findings:**

A business-as-usual scenario shows that the cumulative fiscal space across the 20 countries is US$12.179 billion, which would likely increase by 28% to US$15.612 billion by 2026 but varies across countries—the highest proportional increases expected in Seychelles (117%) and Mozambique (69%) but lowest in Zambia (15%). Under optimistic assumptions, allocating an additional 1.5% of gross domestic product (GDP) to health even without further prioritising the proportional allocation to the wage bill could boost the cumulative fiscal space for HWF by US$4.639 billion. In a very optimistic scenario of a 1.5% increase in health expenditure as a proportion of GDP and further prioritisation of HWF within the health expenditure, the cumulative fiscal space for HWF could improve by some 105%—ranging from 24% in Zambia to 330% in Lesotho.

**Conclusion:**

Small increments in government health expenditure and increased prioritisation of HWF in funding in tandem with the 57% global average could potentially increase the fiscal space for HWF by at least 32% in 11 countries. Unless the HWF is sufficiently prioritised within the health expenditures, only increasing the overall health expenditure to even recommended levels would still portend severe underinvestment in HWF amid unabating shortages to deliver health services. Thus, HWF strategies and investment plans should include fiscal space analysis to deepen advocacy for sustainable investment in the HWF.

**What is already known on this topic**

- Africa faces a significant shortage of 6.1 million health workers, which requires more than 50% of all health system-related investments in education, employment and retention.
- The HWF is a worthwhile investment that guarantees a massive return on investment at a ratio of 1:10, but it remains with chronic underinvestment, hence the need for fiscal space evidence for sustained advocacy toward more significant and smarter investments.
- Most countries also face a new paradigm of health workforce (HWF) challenges where trained health workers remain unemployed for years due to limited fiscal space to absorb them into the health system.

**What this study adds**

- This study provides a blueprint for estimating fiscal space for HWF that will guide the development of investment cases for HWF development, employment and retention.
- Twenty countries in Eastern and Southern Africa in 2021 had a cumulative fiscal space of US$12.179 billion for employing health workers in the public sector, which under a business-as-usual fiscal growth would likely be an increase of 28% by 2026; even under the most optimistic scenarios, the cumulative fiscal space for the countries would increase by 38% in 2026.
- Nine out of 11 (82%) countries (with comparable data) have fiscal space deficits ranging from 15% in Zambia to 87% in Lesotho if all trained health workers currently in the country were employed—the average fiscal deficit is 43% without the private sector contribution. However, the deficit could be reduced to 37%, including the private sector.

**How this study might affect research, practice or policy**

- If the HWF is not sufficiently prioritised (to match the global average of 57%) within government health expenditures, increasing the overall health budget may not inure to sufficient fiscal space needed to address the chronic underinvestment that has fuelled longstanding shortages of HWF.
- It is imperative to include fiscal space analysis in HWF analytics, especially health labour market analysis and HWF planning scenarios.

## Introduction

The global sustainable development agenda places enormous responsibility on health systems to ensure healthy lives and the well-being of all people of all ages.^1^ The attainment hinges on smart investments into health service delivery, with the health workforce (HWF) playing a critical part. It is against this backdrop that the Sustainable Development Goals (SDGs) 3 target (3c) recommends an ‘increase in health financing […] in the recruitment, development, training, and retention of the health workforce … ’.^2^ However, one challenge in the African context is chronic underinvestment in the HWF, resulting in longstanding shortages of health workers, poor working conditions and suboptimal retention. Although recent estimates project a deficit of 6.1 million health workers by 2030 in Africa, the current trends of investment are projected to yield employment (or demand) of only 2.4 million health workers vis-à-vis the potential supply of 3.1 million.^3–6^ Thus, nearly 23% of trained health workers ready to provide health services may fail to find appropriate jobs by 2030 if investments are not made to expand decent employment opportunities for the HWF in Africa.^3^ The Global Strategy on Human Resources for Health: Health Workforce 2030^4^ determined a threshold of 44.5 doctors, nurses and midwives per 10 000 as necessary for the attainment of critical targets of universal health coverage and the SDGs.^6^ A recent report shows that in the African region, only 4 out of 47 countries (Seychelles, Mauritius, Namibia and South Africa) met this threshold by 2018.^7^ The situation appears to be worsening in the context of the immeasurable impact of COVID-19 on the HWF and the bleak economic prospects facing countries in the short-to-medium term, occasioned by the protracted COVID-19 pandemic.^8^

Although the health, social and economic return on investing in the HWF is well documented,^9^ unlocking the potential fiscal space (predominantly from domestic sources) to expand HWF investments in education, employment and retention remains an intricate challenge for many countries in Africa, in the face of many competing investment priorities. The lack of context-specific evidence on what is feasible is one of the limiting factors for sustained policy dialogue toward improving HWF financing through sustainable expansion in the fiscal space for HWF. Thus, the starting point of the advocacy efforts for a sustainable increase in HWF investments is to establish the potential fiscal space.^10^ In an attempt to partly address the evidence gap, this paper draws on existing methodological frameworks and publicly available datasets to estimate the potential fiscal space for HWF across 20 countries in the East and Southern Africa subregion.

### Defining fiscal space for the HWF

Broadly, fiscal space is defined as ‘the capacity of government to provide additional budgetary resources for the desired purpose without any prejudice to the sustainability of the government’s financial position’.^11^ Extending this definition, Tandon and Cashin (piii) explained that ‘ … fiscal space [assessment] typically entails an examination of whether and how a government could feasibly increase its expenditure in the short-to-medium term [in a sector or area], in a way that is consistent with a country’s macroeconomic fundamentals’. For this analysis, we draw on this definition to define fiscal space for the HWF as the ability of governments to direct resources toward HWF investments without unduly compromising the short-to-medium term ability of the government in other functions—substantially crowding out expenditure in other areas of the health sector or other sectors.

We define fiscal space for the health workforce as the ability of governments to direct resources towards health workforce investments without unduly compromising the short-to-medium ability of the government in other functions or substantially crowding out expenditure in other areas of the health sector or other sectors.

## Methods

### Empirical framework for assessing fiscal space: an adaptation for the HWF

Extending the works of Heller,^11^ Tandon and Cashin^12^ developed an empirical framework for analysing fiscal space for priority expenditures of government (equation 1), which was adapted for analysing the fiscal space for HWF. The government’s ability to spend on health (and all other sectors) depends on its tax revenue, supplemented by borrowing, development aid and others. The quantity of tax revenue that can be collected is also a function of the overall size of the economy (ie, gross domestic product (GDP)) and the tax collection ability of the government.

From equation 1, healthcare expenditure is allocated from the G_t_ component—the government’s non-interest expenditure (general government spending), including all social services. Beyond the macroeconomic constraints on the government’s ability to spend, the fiscal space for health also depends on the level of prioritisation given to the health sector within the general government spending.^12–14^ Thus, general government health expenditure (GGHE) in any given year is a proportion K_t_ of the general government expenditure G_t_ (equation 2)—a metric often reported in health expenditure statistics, but whether it is a constant or variable parameter is a context-specific policy question. For instance, if G_t_ increases due to increases in GDP and tax revenues, health spending would increase by a fixed proportion, K_t_, if the spending priorities remain unchanged. On the other hand, health spending could be improved through ‘reprioritisation’, whereby decision-makers explore ways to increase the proportion of K_t_ irrespective of whether G_t_ is changed or not.^12 14^ GGHE can be expressed proportionally to the GDP (equation 3).

Box 1 **Formulae for fiscal space estimation**

$$G_{t}+þ\gamma tB_{t-1}=T_{t}+B_{t}+A_{t}+O_{t}$$

The left side of the equation represents expenditures or budgetary allocations, and the right side represents the generation of resources, where:

- G_t_ is government non-interest expenditure in time t.
- γtB_t−1_ is non-discretionary debt interest payments.
- T_t_ is tax revenue.
- B_t_ is total government borrowing.
- A_t_ is external grants or aid.
- O_t_ is other source of funds (ie, non-tax revenue and cutting lower priority spending).

$$GGHE_{t}=K_{t}\times G_{t}$$

Where:

- GGHE_t_ is GGHE at time t.
- K_t_ is the proportion of overall government expenditure allocated to health.
- G_t_ is the overall government expenditure at time t.

The GGHE can also be expressed as a proportion of the GDP as follows:

$$GGHE_{t}=GDPvalues_{t-1}\times P_{t-1}$$

Where:

- P is the GGHE as a percentage of GDP.

Given that GEHWF is a part of the GGHE, it is expressed as:

$$GEHWF_{t}=GGHE_{t}\times q_{t}$$

Where:

- GEHWF_t_ is GEHWF at time t.
- GGHE_t_ is the GGHE at time t.
- q_t_ is the proportion of GGHE allocated to the HWF at time t.

Alternatively, substituting GGHE in equation 4 in equation 3, GEHWF can be expressed in terms of the GDP as:

$$GEHWF_{t}=GDPvalues_{t-1}\times P_{t-1}\times q_{t}$$

Where:

- GEHWF_t_ is the GEHWF at time t (the fiscal space for HWF).
- P_t–1_ is the GGHE as a percentage of GDP at time t−1.
- q_t_ is the proportion of GGHE allocated to the HWF at time t.

GDP, gross domestic product; GEHWF, government expenditure on the health workforce; GGHE, general government health expenditure; HWF, health workforce. Source: adapted from Tandon and Cashin.^12^

Analogously, the fiscal space for HWF or the Government expenditure on the HWF (GEHWF), usually on wages and remuneration, in a country depends not only on the size of GGHE but also on the level of prioritisation given to the HWF employment within the GGHE. Thus, GEHWF is a proportion, q_t_, of the GGHE (equation 4). There is also no normative standard on the optimal proportion of government health spending that must be spent on the HWF (wage bill). The proportion also depends on the political economy of the health labour market and other contextual and dynamic factors that vary from one country to another. Box 1 provides simple mathematical expressions of the relationships described.

### Data sources used in HWF fiscal space analysis for East and Southern Africa countries

In estimating the available and anticipated fiscal space for HWF across the East and Southern Africa countries, publicly available data from reputable international databases and governments’ public documents, where available, were used. Table 1 provides the definition of the main parameters and the sources from which data was obtained for the analysis.

**Table 1:** Definitions of parameters and data sources

Indicator name	Definition	Year	Source
GDP per capita, current prices (US$)	GDP is expressed in current US$ per person. Data are derived by first converting GDP in national currency to US$ and dividing it by the total population	2010–2018 and projections up to 2026	IMF
GGHE (% GGE)	GGE includes consolidated direct outlays and indirect outlays (eg, subsidies to producers and transfers to households), including the capital of all levels of government, social security institutions, autonomous bodies and other extra-budgetary funds. GGE on health comprises the direct outlays earmarked for the enhancement of the health status of the population and/or the distribution of medical care goods and services among the population by the following financing agents: central/federal, state/provincial/regional and local/municipal authorities; extra-budgetary agencies and social security schemes; and parastatals. All can be financed through domestic funds or external resources	2010–2019	WHO: Global Health Expenditure Database
General Government Expenditure (% GDP)	GGE includes consolidated direct outlays and indirect outlays, such as subsidies and transfers, including capital, of all levels of government social security institutions, autonomous bodies and other extra-budgetary funds	2010–2019	WHO: Global Health Expenditure Database
Current expenditure on health as % of GDP	This indicates the level of health system expenditure within a country relative to the output of the whole country. It shows the importance of the health sector in the economy. Also, it indicates the societal priority of health since the indicator provides information on the level of resources channelled to health relative to other uses in the overall economy	2010–2018	WHO: Global Health Expenditure Database
GGHE as % GGE	Expressing domestic GGHEs as a share of the GGE is essential to compare the size of current public health expenditures relative to the total size of government expenditure	2010–2018	WHO: Global Health Expenditure Database

GDP, gross domestic product; GGE, general government expenditure; GGHE, general government health expenditure; IMF, International Monetary Fund.

### Fiscal space scenarios modelled and their corresponding assumptions

In modelling the potential fiscal space for HWF, three scenarios were simulated primarily based on projected GDP rates, supported by evidence from literature and country-specific reports. The three scenarios are:

1. ‘Business-as-usual scenario’: This scenario assumed that in the future (up to 2026), GGHE as a proportion of GDP will remain similar to the average observed between 2010 and 2019. Therefore, future fiscal space for health and the HWF will be determined solely by GDP growth. It further assumes that the proportion of GGHE spent on the HWF will remain constant at the prevailing levels. Thus, this scenario makes no assumptions that the level of prioritisation of health within the overall government spending or that of the HWF spending within the mix of health spending will change in the medium term. Hence, expansion in the fiscal space for HWF will only be a function of the size of GGHE, which will also be dependent on the macroeconomic conditions.
2. ‘Optimistic scenario’ (fiscal growth and priority increase in health spending): Based on recent evidence about a likely increase in government’s spending on health by an average of 1.5% per annum (range: 0.6%–2.4% per annum) up to 2040 across sub-Saharan Africa,^15^ this scenario assumed that the GGHE as a proportion of GDP will be increased by 1.5% throughout the horizon of the projection. This scenario assumes that the proportion of GGHE spent on HWF will remain constant at the prevailing levels, similar to the baseline or business-as-usual scenario. Thus, while this scenario assumes that the level of prioritisation of health within the overall government spending will increase, that of the HWF spending (in proportional terms) within the mix of health spending will not change.
3. ‘Very optimistic scenario’ (fiscal growth, priority in health and HWF spending): Building on the previous scenario, this scenario assumed that the GGHE as a proportion of GDP will be increased by an average of 1.5% over the period of projection^15^ and that the proportion of GGHE spent on HWF will be increased to the global average of 57%^10^ if a country is not already at that level or higher. This assumption was informed by previous work on fiscal space for HWF globally.^10^

## Results

### Estimated fiscal space for health workforce investments under different scenarios

The ‘business-as-usual scenario’ shows that at baseline, the cumulative fiscal space across the 20 countries was estimated to be US$12.179 billion, which could increase by US$ 3.433 billion (or 28.2%) to US$15.612 billion by 2026 (table 2). The average overall fiscal space per year across the 20 countries is about US$14 billion. Proportionally, South Africa has the largest share of the cumulative fiscal space (68.9%, annual average fiscal space of US$ 9.60 billion), followed by Kenya (8.2%, annual average fiscal space of US$1.54 billion), United Republic of Tanzania (3.3%, annual average fiscal space of US$461 million), Botswana (3.3%, annual average fiscal space of US$455.56 million) and Uganda (2.3%, annual average fiscal space of US$329.1 million). Countries with the least share are South Sudan (0.13%, annual average fiscal space of US$18.84 million), Eritrea (0.06%, annual average fiscal space of US$8.31 million) and Comoros (0.04%, annual average fiscal space of US$5.4 million).

**Table 2:** Estimates of annual public sector fiscal space for HWF under various scenarios in 20 countries, 2022–2026

Country	Projected fiscal space for HWF (US$ million): ‘business-as-usual’						Projected fiscal space for HWF (US$ million): ‘optimistic’						Projected fiscal space for HWF (US$ Million) – ‘very optimistic’					
	Baseline	2022	2023	2024	2025	2026	Baseline	2022	2023	2024	2025	2026	Baseline	2022	2023	2024	2025	2026
Botswana	370.38	398.29	427.84	453.35	481.97	516.35	370.38	404.26	440.77	474.06	511.55	556.26	370.38	499.85	544.99	586.15	632.50	687.78
Comoros	4.56	4.84	5.10	5.39	5.68	6.01	4.56	4.92	5.25	5.63	6.03	6.47	4.56	4.92	5.25	5.63	6.03	6.47
Eritrea	6.88	7.39	7.82	8.28	8.77	9.29	6.88	7.50	8.06	8.66	9.31	10.01	6.88	9.51	10.21	10.97	11.79	12.68
Eswatini	77.69	80.28	82.78	86.31	90.11	98.51	77.69	81.48	85.28	90.25	95.64	106.13	77.69	81.48	85.28	90.25	95.64	106.13
Ethiopia	360.76	356.70	391.47	436.22	481.87	532.91	360.76	362.05	403.31	456.14	511.44	574.10	360.76	458.59	510.85	577.78	647.82	727.19
Kenya	933.46	992.52	1064.58	1145.78	1235.91	1334.71	933.46	1007.41	1096.76	1198.11	1311.75	1437.86	933.46	1400.54	1524.76	1665.67	1823.65	1998.98
Lesotho	24.84	26.69	28.35	29.53	30.77	32.56	24.84	27.09	29.21	30.88	32.66	35.08	24.84	82.58	89.02	94.13	99.56	106.93
Madagascar	156.35	169.35	183.25	196.41	209.69	223.63	156.35	171.89	188.79	205.38	222.55	240.91	156.35	217.73	239.13	260.15	281.90	305.15
Malawi	131.86	136.56	141.28	147.13	153.36	161.96	131.86	138.61	145.55	153.85	162.77	174.47	131.86	154.91	162.68	171.95	181.92	195.00
Mauritius	173.65	190.40	200.17	207.60	214.96	223.25	173.65	193.26	206.22	217.08	228.15	240.51	173.65	193.26	206.22	217.08	228.15	240.51
Mozambique	110.18	117.53	131.32	150.62	172.99	186.25	110.18	119.30	135.29	157.50	183.60	200.65	110.18	151.11	171.36	199.50	232.57	254.15
Namibia	128.39	135.63	141.94	148.10	154.61	161.57	128.39	137.67	146.23	154.86	164.09	174.05	128.39	270.59	287.41	304.39	322.53	342.11
Rwanda	68.69	72.92	80.27	87.98	96.36	104.22	68.69	74.01	82.70	92.00	102.28	112.27	68.69	150.67	168.35	187.29	208.21	228.56
Seychelles	18.82	20.11	23.81	27.94	34.07	40.78	18.82	20.42	24.53	29.21	36.16	43.94	18.82	21.55	25.89	30.84	38.17	46.38
South Africa	8596.96	8978.12	9258.49	9548.05	9850.18	10 616.43	8596.96	9112.79	9538.33	9984.19	10 454.63	11 436.91	8596.96	9112.79	9538.33	9984.19	10 454.63	11 436.91
South Sudan	14.02	16.80	17.78	18.94	19.07	21.62	14.02	17.05	18.32	19.80	20.24	23.30	14.02	21.60	23.21	25.08	25.63	29.51
Uganda	271.64	288.73	306.35	326.54	349.62	374.26	271.64	293.06	315.61	341.46	371.07	403.18	271.64	407.43	438.78	474.71	515.88	560.52
United Republic of Tanzania	372.20	404.29	415.50	448.49	493.47	544.50	372.20	410.36	428.05	468.97	523.75	586.59	372.20	410.36	428.05	468.97	523.75	586.59
Zambia	212.22	213.65	219.30	227.21	235.48	244.22	212.22	216.86	225.93	237.59	249.93	263.09	212.22	216.86	225.93	237.59	249.93	263.09
Zimbabwe	145.79	164.11	163.87	167.55	172.59	179.08	145.79	166.57	168.82	175.21	183.18	192.92	145.79	271.28	274.93	285.34	298.32	314.19
Total	12 179.35	12 774.94	13 291.28	13 867.41	14 491.54	15 612.13	12 179.35	12 966.56	13 693.00	14 500.85	15 380.79	16 818.69	12 179.35	14 137.60	14 960.65	15 877.65	16 878.58	18 448.81

HWF, health workforce.

Although South Africa alone accounts for 69% of the cumulative fiscal space across the 20 countries (US$10.616 billion in 2026), the largest proportional increase over the 5 years under the business-as-usual scenario will likely be in Seychelles (117%), Mozambique (69%), South Sudan (54%) and Rwanda (52%), with the lowest proportional increase in South Africa (23%), Zimbabwe (23%) and Zambia (15%). Economic growth would be the primary driver of potential uncertainty in the projected fiscal space. The upper-middle-income and high-income countries (Botswana, Mauritius, Namibia, Seychelles and South Africa) together will contribute 74.9% (US$ 52.45 billion out of the total of US$70.037 billion) of the 5-year cumulative fiscal space in the East and Southern Africa subregion (table 3).

**Table 3:** Analysis by income group

Scenario	Year(s)	Projected fiscal space by income group			Overall
		Low income	Lower middle income	Upper middle income and high income	
Projected fiscal space for HWF (US$ million)—business-as-usual scenario	Baseline (2021)	1198.07	1693.07	9288.21	12 179.35
	2022	1246.26	1806.11	9722.56	12 774.94
	2023	1342.34	1896.69	10 052.25	13 291.28
	2024	1458.43	2023.95	10 385.04	13 867.41
	2025	1581.84	2173.90	10 735.80	14 491.54
	2026	1712.65	2341.09	11 558.39	15 612.13
	5-year cumulative	7341.51	10 241.74	52 454.03	70 037.28
Projected fiscal space for HWF (US$ million)—optimistic scenario	Baseline (2021)	1198.07	1693.07	9288.21	12 179.35
	2022	1264.96	1833.21	9868.40	12 966.56
	2023	1382.91	1954.01	10 356.08	13 693.00
	2024	1525.05	2116.40	10 859.41	14 500.85
	2025	1678.90	2307.30	11 394.59	15 380.79
	2026	1845.01	2522.02	12 451.67	16 818.69
	5-year cumulative	7696.82	10 732.93	54 930.14	73 359.90
Projected fiscal space for HWF (US$ million)—very optimistic scenario	Baseline (2021)	1198.07	1693.07	9288.21	12 179.35
	2022	1653.03	2386.53	10 098.04	14 137.60
	2023	1809.86	2547.95	10 602.85	14 960.65
	2024	1997.68	2757.33	11 122.64	15 877.65
	2025	2201.36	3001.24	11 675.98	16 878.58
	2026	2418.88	3276.25	12 753.68	18 448.81
	5-year cumulative	10 080.81	13 969.30	56 253.19	80 303.30
Comparison of change in potential fiscal space between the different scenarios	Business-as-usual scenario vs optimistic scenario	43.3%	28.5%	24.1%	
	Business-as-usual scenario vs very optimistic scenario	345.0%	349.7%	155.8%	
	Optimistic scenario vs very optimistic scenario	287.7%	306.7%	125.8%	
	Business-as-usual scenario as % of GDP	0.6%	0.8%	2.7%	
	Optimistic scenario as % GDP	0.6%	0.8%	2.7%	
	Very optimistic scenario as % of GDP	0.6%	0.8%	2.7%	
	% change in fiscal space business-as-usual scenario	43.1%	31.7%	46.8%	
	% change in fiscal space optimistic scenario	54.2%	41.9%	58.1%	
	% change in fiscal space very optimistic scenario	105.1%	113.9%	94.0%	

Note: Low-income countries (Ethiopia, Eritrea, Eswatini, Madagascar, Malawi, Mozambique, Rwanda, South Sudan and Uganda), lower-middle-income countries (Comoros, Kenya, Lesotho, United Republic of Tanzania, Zambia and Zimbabwe) and upper-middle-income and high-income countries (Botswana, Mauritius, Namibia, Seychelles and South Africa).

GDP, gross domestic product; HWF, health workforce.

In the ‘optimistic scenario’ (which assumes that economies will grow as projected by the International Monetary Fund and that health expenditure as a proportion of GDP will increase by 1.5%), the cumulative fiscal space could increase by 38.1% from US$12.179 billion at baseline to US$16.818 billion by 2026, with an average annual fiscal space of US$14.672 (table 2). Thus, increasing health spending as a proportion of GDP by an average of 1.5% even without further prioritising, the proportional allocation to the wage bill could boost the fiscal space by US$4.639 billion across the 20 countries. Under this scenario, South Africa would have the largest share (69% average annual fiscal space of US$10.107 billion) of the anticipated cumulative fiscal space, followed by Kenya (8.2%, average annual fiscal space of US$1.21 billion), Botswana (average annual fiscal space of US$477.38 million) and United Republic of Tanzania (average annual fiscal space of US$344.88 million), each with a share of 3.3%. In the scenario, the average proportional increase in fiscal space across all the countries within 5 years (2022–2026) is about 51%, ranging from 24% in Zambia to 133% in Seychelles. Upper-middle-income and high-income countries will have the largest proportional increase by 58%, followed by low-income countries (54%) and lower-middle-income countries (48%) (table 3).

The ‘very optimistic scenario’ suggests that the cumulative fiscal space for the HWF across the 20 countries could increase by US$6.269 billion from the baseline of US$12.179 billion in 2021 to US$16.878 billion by 2026 (table 4). Thus, this scenario would likely yield an annual average fiscal space of US$16.06 billion across the 20 countries. Across income groups, upper-middle-income and high-income countries will contribute 70.1% to the overall fiscal space (amounting to US$11.250 billion), followed by lower-middle-income countries (17.4%, US$2.794 billion) and low-income countries (12.6%, US$2.016 billion) (table 3).

**Table 4:** Comparison of the different fiscal space projection scenarios: business-as-usual vs optimistic vs very optimistic scenarios

Country	5-year projected fiscal space for HWF (US$ Million) – ‘business as usual scenario.’	5-year projected fiscal for HWF (US$ Million) – ‘optimistic scenario’	5-year projected fiscal space for HWF (US$ Million) – ‘very optimistic scenario’	Comparison of change in potential resource availability between the different scenarios (from baseline)		
				‘Business as usual scenario’ vs ‘Optimistic scenario’	‘Business as usual scenario’ vs ‘Very Optimistic scenario’	‘Optimistic scenario’ vs ‘Very Optimistic scenario’
Botswana	2277.81	2386.90	2951.27	4.79%	29.57%	23.64%
Comoros	27.02	28.31	28.31	4.76%	4.76%	0.00%
Eritrea	41.56	43.54	55.15	4.77%	32.71%	26.67%
Eswatini	437.99	458.78	458.78	4.75%	4.75%	0.00%
Ethiopia	2199.17	2307.03	2922.24	4.90%	32.88%	26.67%
Kenya	5773.49	6051.89	8413.60	4.82%	45.73%	39.02%
Lesotho	147.91	154.92	472.22	4.74%	219.26%	204.81%
Madagascar	982.33	1029.53	1304.07	4.80%	32.75%	26.67%
Malawi	740.29	775.26	866.47	4.72%	17.04%	11.76%
Mauritius	1036.39	1085.22	1085.22	4.71%	4.71%	0.00%
Mozambique	758.71	796.33	1008.68	4.96%	32.95%	26.67%
Namibia	741.84	776.91	1527.02	4.73%	105.84%	96.55%
Rwanda	441.76	463.26	943.07	4.87%	113.48%	103.57%
Seychelles	146.72	154.26	162.83	5.14%	10.98%	5.56%
South Africa	48 251.28	50 526.85	50 526.85	4.72%	4.72%	0.00%
South Sudan	94.21	98.71	125.03	4.77%	32.71%	26.67%
Uganda	1645.50	1724.38	2397.31	4.79%	45.69%	39.02%
United Republic of Tanzania	2306.25	2417.72	2417.72	4.83%	4.83%	0.00%
Zambia	1139.86	1193.39	1193.39	4.70%	4.70%	0.00%
Zimbabwe	847.21	886.71	1444.06	4.66%	70.45%	62.86%
ESA	70 037.28	73 359.90	80 303.30	4.80%	42.53%	36.01%

Across the individual countries, this scenario could potentially improve the fiscal space for HWF by some 51.47% over 5 years, ranging from 24% (US$32 million) in Zambia to 330% (US$7.72 million) in Lesotho. As this scenario had a further assumption that countries would commit at least 57% of their health budget to the HWF, 14 countries (Botswana, Eritrea, Eswatini, Ethiopia, Kenya, Lesotho, Malawi, Namibia, Rwanda, Seychelles, South Sudan, Uganda, United Republic of Tanzania and Zimbabwe) would have enhanced fiscal space for HWF (over and above the previous scenario) with this very optimistic assumption. It would, however, seem a very tall order as most countries are instead on a drive to limit recurrent expenditures related to wages.

### Comparing the three scenarios: ‘business as usual’ versus ‘optimistic’ vs ‘very optimistic’

As shown in table 4, the cumulative 5-year fiscal space for HWF ranges between US$70.037 billion in the ‘business-as-usual scenario’ and US$80.303 billion in the ‘very optimistic scenario’. Comparing the ‘business-as-usual scenario’ with the ‘optimistic scenario’ suggests a potential for expanding the HWF fiscal space in all the 20 countries with a 5-year average incremental expansion of 4.8% (range: from 4.66% in Zimbabwe to 5.14% in Seychelles).

Also, across all the countries, the ‘very optimistic scenario’ compared with the ‘business-as-usual scenario’ could expand the cumulative fiscal space by an average of 42.53% by 2026, which could be as high as 219.26% in Lesotho. In 12 out of 20 countries (namely, Botswana Eritrea, Ethiopia, Kenya, Lesotho, Madagascar, Mozambique, Namibia, Rwanda, South Sudan, Uganda, and Zimbabwe), they could increase the HWF fiscal space by at least 25% if the ‘very optimistic scenario’ was pursued.

Compared with the ‘optimistic scenario’, the ‘very optimistic scenario’ could increase 36% in the cumulative fiscal space across the 20 countries. However, in 6 out of the 20 countries (30%) (namely, Comoros, Eswatini, Mauritius, South Africa, United Republic of Tanzania and Zambia), where the HWF already consumes at least 57% of the government health spending, even the ‘very optimistic scenario’ offers no room for fiscal expansion for HWF compared with the ‘optimistic scenario’. Of note, countries like Madagascar and Comoros are said to be, respectively, spending as high as 84% and 90% of their health expenditure on HWF (see online supplemental material); hence, there will be little or no room for HWF fiscal expansion.

10.1136/bmjgh-2021-008416.supp1 Supplementary data

### Estimated cost of employing existing health workers compared with baseline fiscal and financial space in 11 countries

In 11 countries, there was data on the average income of health workers and the existing stock of health workers from public and private sectors. For these countries, the total cost of salaries (assuming all the health workers were to be employed) was computed and compared with the estimated fiscal space. As shown in table 5, the cumulative cost of employment for health workers in the 11 countries was US$19.286 billion, which was highest in South Africa (US$ 14.43 billion or 75% of the total) and lowest in Eswatini (US$ 78.62 million or 0.41% of the total). Kenya’s cost of wages was US$1.626 billion (8.4% of the total), and that of Ethiopia was estimated to be US$ 864.63 million (4.5% of the total), similar to US$ 875.93 million in Namibia. Overall, an average of 3.2% of GDP was required to employ and/or sustain the jobs of all trained health workers in these countries. This, however, varied from 0.8% of GDP needed in Ethiopia and Malawi to as much as 10% and 8.2% required in Lesotho and Namibia, respectively.

**Table 5:** Estimated cost of wages of health workers compared with total baseline fiscal and financial space in 11 countries (US$ million)

No.	Country	General practitioners	Specialist medical doctors	Nurses and midwives	Dentists	Pharmacists	Laboratory scientists and technicians	Community health workers	Other health workers*	Total	Total as % of 2020 GDP (current US$)	Baseline fiscal space	Fiscal gap (public sector only)	% of fiscal gap	Baseline financial space	Financial gap (including private sector)	% of financial gap
1	Botswana	32.87	2.61	207.52	3.79	16.47	15.56	n.d.	21.24	300.06	1.9%	370.38	70.32	23%	382.25	82.19	27%
2	Eswatini	5.54	2.14	24.06	1.94	4.98	3.33	1.90	34.74	78.62	2.0%	77.69	(0.94)	−1%	86.06	7.44	9%
3	Ethiopia	25.52	29.09	236.17	8.22	41.73	31.67	98.89	393.34	864.63	0.8%	360.76	(503.87)	−58%	487.39	(377.24)	−44%
4	Kenya	214.67	121.51	718.96	67.60	75.18	n.d.	13.94	413.91	1625.76	1.6%	933.46	(692.30)	−43%	1159.52	(466.25)	−29%
5	Lesotho	23.39	6.21	36.91	4.56	7.15	2.50	31.78	72.86	185.36	10.0%	24.84	(160.52)	−87%	29.00	(156.36)	−84%
6	Malawi	15.55	2.60	32.64	0.61	2.10	2.94	20.13	20.22	96.78	0.8%	131.86	35.08	36%	146.43	49.65	51%
7	Namibia	69.99	28.81	656.25	18.79	41.45	4.71	4.90	51.03	875.93	8.2%	128.39	(747.54)	−85%	138.63	(737.29)	−84%
8	Rwanda	8.73	10.38	49.74	1.05	1.28	9.20	96.22	2.33	178.94	1.7%	68.69	(110.25)	−62%	76.23	(102.71)	−57%
9	South Africa	2055.63	1320.72	9481.17	178.07	895.04	n.d.	162.54	337.52	14 430.67	4.8%	8596.96	(5,833.71)	−40%	9262.95	(5,167.72)	−36%
10	Zambia	42.71	12.08	104.55	11.23	14.23	9.33	2.70	159.03	355.85	1.8%	212.22	(143.62)	−40%	236.41	(119.44)	−34%
11	Zimbabwe	50.96	3.09	95.49	9.07	2.78	2.32	4.58	125.82	294.10	1.8%	145.79	(148.31)	−50%	180.22	(113.88)	−39%
	Total	2545.56	1539.25	11 643.45	304.92	1102.39	81.55	437.59	1632.02	19 286.72	3.2%	11 051.05	(8,235.66)	−43%	12 185.11	(7,101.61)	−37%

n.d.: no data on the existing stock of the cadre of health professionals in the country; hence, the cost of wages could not be computed.

Data sources: the number of health workers was taken from WHO/AFRO health workforce survey results; income data were taken from health labour market analysis reports.

*Includes all other health professionals, heath managers and support staff.

Nurses and midwives employment costs constituted about 60% of the total (ranging from 27% in Ethiopia to 75% in Namibia), followed by general practitioners (13.25%), medical specialists (8%) and other workers (8.5%).

Compared with the public sector fiscal space, the cost of employment showed an aggregate deficit of 43%. All the countries except Botswana and Malawi had deficits ranging from 1% in Eswatini to 87% in Lesotho. When the estimated fiscal space was adjusted for the private sector contribution, the overall deficit decreased from 43% to 37%. Even with the private sector contribution taken into account, only three countries (Botswana, Eswatini and Malawi) could potentially absorb all the existing stock of health workers in 2020 under the prevailing levels of health worker remuneration.

## Discussion

This paper draws on existing methodological frameworks and publicly available datasets to estimate the potential fiscal space for HWF across 20 countries in the Eastern and Southern Africa subregion. Increasing the fiscal space for HWF investments would require some or all of (a) expanding the overall health expenditure through reprioritising health within the overall government expenditure, for example, meeting the Abuja target, (b) increasing the prioritisation of the HWF within the health expenditure and (c) earmarking funds for HWF investments from innovative financing initiative such as specific health taxes from alcohol, sugar and vehicle insurance among others.

The analysis revealed that 60% of the countries (n=12) could increase the fiscal space for HWF by at least 25% if the government’s health expenditure was increased by at least 1.5% of GDP and at least 57% (the global average) was prioritised for the HWF—the ‘very optimistic scenario’ in the analysis. In three countries (Lesotho, Namibia and Rwanda) with HWF expenditure of less than 30% of the government health expenditure, the scope for even larger expansion in the HWF fiscal space seemed plausible. However, given the substantial economic shocks experienced by countries due to the COVID-19, and the anticipated slow recovery,^16–18^ the macroeconomic outlook suggests a minimal potential for increased public expenditure in the short-to-medium term. Our analysis shows that maintaining the prevailing level of prioritisation and relying on GDP growth to eventually reflect in expanded fiscal space for HWF (business-as-usual scenario) would be insufficient to absorb all the trained health workers—there will be a fiscal deficit of 43%, ranging from 1% to 87%. Although there is no ‘gold standard’ on the proportion of government health expenditure that must be spent on the HWF, previous analysis of data from 136 countries showed an average of 57%, but 60% is often assumed for analytical purposes.^10^ Also, a forecast of the resource requirements for attaining the health-related SDGs revealed that at least 50% of all the additional funding needed must be spent on HWF education and employment.^19^ As supported by this analysis, unless the HWF is sufficiently prioritised (to at least the global average of 57%) within the government health expenditure, only increasing the overall health budget to recommended levels such as the Abuja target may not yield sufficient fiscal space needed to address the chronic underinvestment that has fuelled longstanding shortages of HWF.

In Zimbabwe, for example, a fiscal space analysis for health conducted in 2017 by the World Bank suggested that with the macroeconomic trajectory at the time, per capita public spending on health could slightly decrease to US$ 27 per capita in 2020, compared with US$ 28 per capita in the preceding years between 2012 and 2014.^20^ Also, in United Republic of Tanzania, a fiscal space analysis by UNICEF in 2017 concluded that based on baseline status quo scenarios, where economic growth is in line with recent trends, increasing spending on priority sectors based on projected needs could lead to fiscal deficits.^21^ Similarly, a health labour market analysis recently conducted in Ethiopia, Lesotho and Kenya pointed to budgetary and financial space gaps to address existing HWF unemployment.^22–24^ Thus, the direction of evidence from this analysis appears to be consistent with recent fiscal space analyses in some of the countries in East and Southern Africa.

As part of the legacy of the structural adjustment programme of the 1990s,^25–27^ many African countries have arbitrary but rigid wage bill ceilings, some of which are enshrined in legislation that makes it extremely difficult to increase the prioritisation of HWF within the health budget.^28–30^ In such context, most decision-makers tend to be overly concerned about controlling escalation in the wage bill as a proportion of the overall health budget by cutting down the wage bill (the numerator) rather than focusing on expanding the health budget (the denominator). In such contexts as Kenya, efforts to increase fiscal space for HWF should focus on expanding the overall health spending toward the Abuja target or at least $86 per capita alongside intensive, evidence-based policy dialogue for legislative re-engineering to relax the wage ceiling in the health sector.

However, in other contexts, such as Comoros, Madagascar, Mauritius, Mozambique, South Africa and Zambia, the HWF already consumes at least 57% of government health spending becomes difficult to justify further prioritisation of the HWF within the health budget. Nevertheless, most of these countries historically allocate less than the ‘Abuja target’ of 15% of general government expenditure to health. In such contexts, policy dialogue to expand the fiscal space for HWF should focus more on opportunities for expansion in the overall health expenditure (within which the HWF component would also increase by a fixed proportion of the current level of prioritisation).

### Main limitations of the analysis

The main limitations of this analysis relate to data and methodological assumptions. It is worth noting that data on the public sector health expenditure that is allocated to the HWF expenditure was not publicly available for Eritrea. In the absence of this data, the global average of the HWF expenditure as a proportion of the government health expenditure for countries in the same income bracket was used as a proxy.^10^ Also, the study did not analyse the potential fiscal space that could be gained through increased efficiency in public spending and health system efficiency gains (technical and allocative efficiency) as the available data did not allow so. Furthermore, it has been assumed that as economies grow and government revenues increase, governments will prioritise health and then HWF investments. However, the actual level of investments is likely to be influenced by competing political priorities; hence, deviations from the projections will not be unexpected. The analysis was entirely based on reliable secondary data, some of which were only available up to 2018; hence, the average of the last observations (most recent years) between 2009 and 2018 was used as the baseline. Finally, there was no reliable data on the private sector contribution across all countries; hence, the analysis was restricted to fiscal space rather than a complete financial space analysis, encompassing the public and private sectors.^31^

## Conclusion

Twenty countries in the East and Southern Africa subregion had a cumulative HWF fiscal space of US$12.179 billion in 2021, which under a business-as-usual scenario could increase by an average of 5.6% per annum up to 2026. If overall health expenditure were increased by 1.5% of GDP, and without increasing the prioritisation of HWF within the health budget of countries (‘optimistic scenario’), the cumulative fiscal space for HWF could increase by 7.6% per annum up to 2026. However, suppose countries increased the health expenditure by 1.5% of GDP and spent at least 57% of their health budgets on HWF investments (‘very optimistic scenario’), the cumulative fiscal space HWF across the 20 countries could increase by 21% per annum up to 2026. Twelve countries (60%) could increase the HWF fiscal space by at least 25% if the ‘very optimistic scenario’ is pursued. The critical lesson is that unless the HWF was sufficiently prioritised within the health expenditure (at least 57%), increasing the overall health budget to recommended levels will still leave the HWF heavily underinvested, increasing unemployment amid unabating shortages with dire consequences for quality health service delivery. Therefore, all HWF strategies and investment plans should include a fiscal space analysis, which should be used to deepen advocacy for sustainable investment in the HWF.

## Data Availability

All data relevant to the study are included in the article or uploaded as supplementary information.
