# Supplementary material for: Investing in the health workforce: fiscal space analysis of 20 countries in East and Southern Africa, 2021–2026
Source: BMJ Glob Health. 2022 Jun 30;7(Suppl 1):e008416. doi: 10.1136/bmjgh-2021-008416 (PMC9247660; doi:10.1136/bmjgh-2021-008416)
Supplement: Supplementary data [file bmjgh-2021-008416supp001.pdf]

Investing in the health workforce: fiscal space analysis of twenty countries in *East and Southern Africa*, 2021 - 2026

James Avoka Asamani*, Jesse Kigozi, Brivine Sikapande, Christmal Dela Christmals, Sunny Okaroafor, Hamza Ismaila, Adam Ahmat, Jennifer Nyoni, Juliet Nabyonga-Orem and Kasonde Mwinga

SUPPLEMENTARY MATERIAL

Macroeconomic indicators

In the ESA region, GDP growth rates averaged 3% in the baseline period of 2019 (Figures S1 & 2) and the average GDP value was approximately US$ 36 billion (Table S2). GDP growth rates are associated with increased government revenues through tax collection. Revenue collection in ESA, is around 19% of GDP based on tax revenue (as Percent of GDP) (Figure 2). Low- and lower-middle-income countries in the ESA region tended to collect less revenue, with for example Ethiopia, Madagascar, Uganda, Zimbabwe respectively collecting 7.5%, 10%, 11.7% and 11.6% of GDP. In contrast, upper-middle-income and high-income countries are collecting an average of 25.82%. For example, between 2016 and 2018, 20.5%,18.9%, 30.5%, 32.6%, 26.6% of GDP were collected as taxes in Botswana, Mauritius, Namibia, Seychelles, and South Africa respectively.

Government expenditure on health

Domestic Government Health Expenditure (GGHE-D) (37%) and Private expenditures (35%) on average comprised the largest share of total healthcare spending. Government spending on health in ESA countries describes the level to which health is prioritised within governments' public expenditure. Government budget allocations can be associated with the level of government revenue and expenditure levels. GGHE-D averaged 2% of GDP (range: 1 – 5%) in 2016-2018. Total healthcare spending in ESA countries averaged 6 Percent of the GDP values.

Current expenditure on health as a percentage of government expenditure was on average 8% (ranging from 2% in Eritrea and South Sudan to 14% in Madagascar). None of the countries have been able to meet the 15% Abuja target of general government expenditure in 2016-2018 with South Africa reaching close to 14% (W. H. WHO, 2016). Most of the countries in this region are still significantly falling short of this target (See Table 1). Apart from Botswana, most of the countries have kept a constant level of funding in relation to GGHE. The trends of the average CHE and GGHE as a Percentage of the GDP are shown in Out of pocket (as Percent of expenditure on health) averaged 24%.

Table S1: Current health expenditure as a proportion of GDP, 2011 - 2018

Country Name	Current health expenditure as a proportion of GDP (%)								Average (Baseline)
	2011	2012	2013	2014	2015	2016	2017	2018	
Botswana	5.78	6.14	6.22	5.60	5.73	5.56	6.13	5.85	5.85
Comoros	5.44	4.67	4.60	4.56	4.74	4.70	4.65	4.59	4.65
Eswatini	8.47	7.82	7.46	7.26	7.03	6.78	6.91	6.54	6.74
Eritrea	5.21	3.78	4.99	4.05	4.46	3.54	3.76	4.09	3.80
Ethiopia	4.47	4.54	4.08	4.03	3.82	3.66	3.50	3.30	3.49
Lesotho	8.80	8.58	9.01	8.65	8.49	8.01	8.95	9.28	8.75
Mauritius	4.31	4.28	4.68	5.49	5.70	5.71	5.72	5.83	5.75
Malawi	7.49	8.48	11.58	9.71	9.33	9.69	9.64	9.33	9.56
Madagascar	5.19	4.41	4.23	5.08	5.72	6.02	5.51	4.79	5.44
Namibia	9.83	9.26	8.67	8.17	9.71	8.86	8.29	7.95	8.37
Rwanda	8.22	8.36	6.97	7.17	6.86	7.14	6.93	7.54	7.21
Seychelles	4.80	5.73	4.51	4.74	4.59	5.18	4.99	5.11	5.09
South Africa	7.50	7.75	7.72	7.93	8.20	8.10	8.11	8.25	8.15
South Sudan	N/A	N/A	N/A	N/A	N/A	N/A	8.37	6.40	7.39
Tanzania	5.06	5.02	4.65	4.01	3.65	3.96	3.64	3.63	3.74
Uganda	8.92	7.87	7.25	6.97	6.79	6.55	6.49	6.53	6.52
Zimbabwe	8.08	6.92	7.11	8.13	7.45	7.65	5.85	4.73	6.08
Zambia	3.46	3.93	4.69	3.83	4.44	4.48	4.40	4.93	4.60
Mozambique	5.40	5.62	6.50	6.58	7.24	7.85	8.14	8.17	8.05
Kenya	5.81	5.61	5.52	5.46	5.22	5.14	4.84	5.17	5.05
**ESA Average**	**6.43**	**6.25**	**6.34**	**6.18**	**6.27**	**6.24**	**6.24**	**6.10**	**6.21**

TABLE S2: Financing Data for 20 ESA countries (averages of 2016 – 2018)

Country	GDP Growth (%)	GDP Values (billion US$)	CHE%GGE	OOP%CHE	DPVTCHE	GGHED%CHE	GGHED%GGE	GGHED as % GDP	GGE%GDP	CHE%GDP	Tax% GDP	Exp% GDP
**Botswana**	3.9	17.5	13	3	20	73	13	4	18.3	6	20.5	32.8
**Comoros**	3.8	1.1	3	74	76	12	3	1	10.5	5		19.3
**Eritrea**	3.5	2.0	2	52	52	18	2	1	0.0	4		33.7
**Eswatini**	1.8	4.3	8	11	25	42	8	3	23.3	7		35.0
**Ethiopia**	8.6	80.4	5	35	45	24	5	1	10.4	3	7.5	16.8
**Kenya**	5.7	82.8	8	24	42	43	8	2	13.0	5	15.7	26.2
**Lesotho**	-0.6	2.3	11	17	17	62	11	5	39.5	9	31.1	54.1
**Madagascar**	3.7	13.4	14	25	30	43	14	2	14.9	5	10.0	14.4
**Malawi**	3.2	6.6	10	11	18	29	10	3	14.2	10	16.9	28.4
**Mauritius**	3.8	13.4	10	49	56	43	10	2	15.3	6	18.9	25.9
**Mozambique**	3.6	13.7	6	10	16	22	6	2	23.6	8	22.7	30.5
**Namibia**	0.0	12.5	11	8	47	46	11	4	26.2	8	30.5	38.4
**Rwanda**	6.2	9.4	9	11	29	32	9	2	15.2	7	14.2	26.3
**Seychelles**	3.6	1.5	10	24	26	72	10	4	21.7	5	32.6	37.1
**South Africa**	0.9	341.3	13	8	44	54	13	4	21.0	8	26.6	33.4
**South Sudan**	-7.1	3.9	2	21	25	10	2	1	0.0	7		40.3
**Uganda**	6.9	55.1	5	39	41	15	5	1	8.2	7	11.7	16.6
**Tanzania**	4.5	33.2	9	23	24	42	9	2	21.8	4	17.3	16.5
**Zambia**	3.8	24.1	7	11	18	39	7	2	15.0	5	15.5	26.9
**Zimbabwe**	3.0	20.6	8	24	50	27	8	2	8.8	6	11.6	19.7

Health workforce expenditure as a percentage of general government health expenditure

Country-level data on the share of HWF expenditure as a proportion of government health expenditure was not publicly available for eight (40%) of the countries: Comoros, Eritrea, Eswatini, Madagascar, Mauritius, Mozambique, South Sudan, Tanzania. For these countries, we interpolated with the global averages for countries within their income group (Lauer et al., 2017). When those without country-level data were excluded, the health workforce expenditure as a share of the GGHE in the ESA region was an average of 43% but ranges from 19% in Lesotho to 62% in Zambia. However, when all countries, including those we interpolated from global averages are included, the average is 47% of the health expenditure spent on the health workforce. About 75% (n = 9) of the countries with country-level data spent at least 30% of their current health expenditure on the health workforce. Only three countries spent less than 30% of their health their health expenditure on the health workforce. See Table S3 for details.

Table S3: Health workforce expenditure as a percentage of general government health expenditure (GGHE).

Country	Estimated GGHE allocated to HWF (%)	latest Year	Source
Botswana	46	2019	Unicef health budget brief 2019
Comoros	90	2015	Unicef Public Expenditure Review 2015
Eritrea	45		Lauer et al (2018) - average for countries in similar income level
Eswatini	39	2013 - 2018	Unicef health budget brief 2018
Ethiopia	45	2019	Ethiopia HLMA, 2020
Kenya	41	2014	Kenya Productvity paper, 2019
Lesotho	21	2017	Lesotho HLMA report, 2021
Madagascar	84	2013	Public expenditure review Education and Health, 2014
Malawi	51	2020	Public expenditure review
Mauritius	57	2022	MOF, Mauritus
Mozambique	58	2009 - 2014	Munir 2018. Doctoral thesis: https://dash.harvard.edu/handle/1/37945643
Namibia	29	2019	Public expenditure review
Rwanda	28	2015	Rwanda HLMA report, 2019
Seychelles	54	2014	Public expenditure review
South Africa	61	2017	Public expenditure review
South Sudan	38	2014	UNDP South Sudan August, 2014
Uganda	41	2018	Public expenditure review
United Republic of Tanzania	43	2020	Public expenditure review
Zambia	62	2018	Public expenditure review
Zimbabwe	35	2020	Zimbabwe's HLMA (2021)
